# AI and Chemistry in Action: Transforming Crystallization for Scalable Water Harvesting Solutions

**DOI:** 10.1021/acscentsci.4c01838

**Published:** 2024-12-25

**Authors:** Zhiling Zheng

Since childhood, I have always been captivated by the wonders of science, even though I had no access to a laboratory during my early education. I liked to envision myself dressing like Albert Einstein in a classic lab coat, standing in front of a long table, gingerly combining strange solutions inside two test tubes, bubbling, and then watching in awe as shiny, colorful crystals formed in the glassware! As the first person in my family to finish high school, my parents did not fully understand what chemistry was, but they always supported my growing interest in experiments. I began exploring crystallization in our kitchen at home, growing table salt, alum crystals, and rock candy. These early, simple experiments fueled my passion for science, particularly crystallization.

I was finally able to see a real laboratory in college. Sighing at how superficial I used to be, while believing being proficient in experiments is everything necessary for a chemist, I told myself to leap forward. Yet, as a first-generation college student, I faced challenges that are all too familiar to others from underrepresented or minority backgrounds: financial barriers, limited guidance, a lack of professional networks, and, at times, self-doubt. Fortunately, my passion and curiosity helped me get through those dark moments. I found invaluable support through first-generation student organizations and was fortunate to gain undergraduate research experience that set me on the path toward graduate school.

As I transitioned into my Ph.D., my journey with crystallization deepened. I focused on metal–organic frameworks (MOFs), a class of materials with vast potential for critical applications like atmospheric water harvesting (AWH). I soon realized that growing crystals was not just about their visual beauty and bigger size; it was about unlocking their potential for solving global challenges, such as water scarcity, which is a pressing issue affecting regions like the Middle East, North Africa, Central Asia, Australia, and the southwestern United States.^2^ In response to this challenge, chemists from various geographic regions, racial backgrounds, and scientific disciplines are coming together to use chemistry as a tool to fight against the same water shortage challenge.^3,4^ I have been fortunate to be part of this global effort and work with people from different backgrounds, using AI tools like large language models (LLMs) for data mining and machine learning models to predict MOF synthesis.^5−8^ These innovations have enabled us to navigate the complexities of MOF crystallization, leading to better-quality frameworks with higher crystallinity.^9,10^ In a recent field test^11^ in Death Valley National Park, our research group demonstrated significant progress—watching water steadily drip into a collection vial under extreme conditions of 51.6 °C and 14% humidity, a powerful step forward in scalable water-harvesting technologies.^12−14^

This work also ties into the future of STEM education and the need for greater inclusivity in science. By exposing students, especially those from underrepresented backgrounds, to cutting-edge research in atmospheric water harvesting, we can foster a new generation of diverse scientists. I have contributed to the design of educational programs that bring MOF crystallization into high school classrooms, allowing students to grow their own MOF crystals and experience the real-world impact of chemistry.^15^ In addition, I have integrated AI tools like LLMs into STEM education, teaching middle school students how to use AI as a digital mentor. This helps provide just-in-time feedback, making advanced chemistry more accessible and engaging for students from all backgrounds. Through these initiatives, I hope to inspire students—regardless of race, gender, or economic background—to discover their passion for chemistry. By nurturing this interest at an early stage, we can help them grow into the scientists and chemists of tomorrow.

I hope that when readers see my cover art and read my story, they will feel empowered. Whether it’s a child growing crystals in their kitchen, a high school student exploring AI, or a young adult pursuing higher education, I want to remind everyone that they are not alone in their journey. Science is for everyone, and the path, while challenging, can also be incredibly rewarding and fun.
